# Are Pain Polymorphisms Associated with the Risk and Phenotype of Post-COVID Pain in Previously Hospitalized COVID-19 Survivors?

**DOI:** 10.3390/genes13081336

**Published:** 2022-07-26

**Authors:** César Fernández-de-las-Peñas, Rocco Giordano, Gema Díaz-Gil, Antonio Gil-Crujera, Stella M. Gómez-Sánchez, Silvia Ambite-Quesada, Lars Arendt-Nielsen

**Affiliations:** 1 Department of Physical Therapy, Occupational Therapy, Rehabilitation and Physical Medicine, Universidad Rey Juan Carlos, 28922 Alcorcón, Spain; silvia.ambite.quesada@urjc.es; 2 Center for Neuroplasticity and Pain (CNAP), SMI, Department of Health Science and Technology, Faculty of Medicine, Aalborg University, DK-9220 Aalborg, Denmark; rg@hst.aau.dk (R.G.); lan@hst.aau.dk (L.A.-N.); 3 Research Group GAMDES, Department of Basic Health Sciences, Universidad Rey Juan Carlos (URJC), 28922 Madrid, Spain; gema.diaz@urjc.es (G.D.-G.); antonio.gil@urjc.es (A.G.-C.); stella.gomez@urjc.es (S.M.G.-S.); 4 Department of Medical Gastroenterology, Mech-Sense, Aalborg University Hospital, DK-9000 Aalborg, Denmark

**Keywords:** single nucleotide polymorphism, COVID-19, pain, post-COVID, risk

## Abstract

Objective: To investigate the association of different, selected pain polymorphisms with the presence of de novo long-COVID pain symptoms and to analyze the association between these polymorphisms with clinical, sensory-related, cognitive and psychological variables in COVID-19 survivors. Methods: Two hundred and ninety-three (*n* = 293, 49.5% female, mean age: 55.6 ± 12.9 years) previously hospitalized COVID-19 survivors participated. Three genotypes of the following single nucleotide polymorphisms (SNPs) were obtained from non-stimulated saliva: *OPRM1* (rs1799971), *COMT* (rs4680), *BDNF* (rs6265), and *HTR1B* (rs6296) by polymerase chain reactions in all participants. Further, clinical (intensity/duration of pain), sensory-related (sensitization-associated symptoms, neuropathic pain features), psychological (anxiety or depressive levels, sleep quality), and cognitive (catastrophizing, kinesiophobia) variables were collected in those COVID-19 survivors suffering from post-COVID pain. Analyses were carried out to associate clinical features with genotype. Results: Participants were assessed 17.8 ± 5.2 months after hospitalization. One hundred and seventeen (39.9%) experienced post-COVID pain (particularly of musculoskeletal origin). The distributions of the genotype variants of any SNP were not significantly different between COVID-19 survivors with and without long-term post-COVID pain (all, *p* > 0.178). No differences in sensitization-associated symptoms, neuropathic pain features, catastrophizing, kinesiophobia levels, anxiety and depressive levels or sleep quality according to the genotype variant in any SNPs were found. No effect of gender was identified. Conclusion: The four SNPs generally associated with pain did not appear to predispose to the development of de novo long-COVID pain symptoms in previously hospitalized COVID-19 survivors. The SNPs were not involved in the phenotypic features of post-COVID pain either.

## 1. Introduction

At the end of 2019, the severe acute respiratory syndrome coronavirus 2 (SARS-CoV-2) first discovered in China, started to spread rapidly to all of Asia and the rest of the world. In March 2020, the World Health Organization (WHO) declared the coronavirus disease-19 (COVID-19) as a pandemic. The features of the COVID-19 pandemic had shown a marked geographical variation with, for example, Western Europe severely affected by the virus, and with a higher death rate in the eastern part of the continent [1]. This evidence can be explained by a potential genetic variation of the host and not by viral mutations. Indeed, genetic polymorphisms, as a consequence of human evolution, allow a population to live in different environments and can be the cause of changes in many plasma proteins and are related to biodiversity, genetic variation, and adaptation [1]. In the case of COVID-19, most genetic studies have focused on the role of the angiotensin-converting enzyme 2 (ACE2) and protease transmembrane protease serine 2 (TMPRSS2) polymorphisms [2]. COVID-19 has led to millions of acute cases and thousands of deaths; healthcare professionals are at the front of a second potential outbreak, the development or persistence of symptoms after the acute phase, a condition called long COVID [3] or post-COVID-19 [4]. Among the symptoms reported by individuals with long COVID, pain prevalence ranges from 4.6 to 23.6% during the first six months after the infection and ranges among the most prevalent symptoms [5]. Some polymorphic candidate genes may play a role in post-COVID pain perception in these individuals. In fact, alterations to several genes and single nucleotide polymorphisms (SNPs) have been proposed to be involved in pain sensitivity [6,7]. Among several gene alterations, the SNPs of *OPRM1* (rs1799971), *COMT* (rs4680), *BDNF* (rs6265), and *HTR1B* (rs6296) have been shown to be associated with different painful conditions and their chronicity [8,9].

Alteration of the µ-opioid receptor gene (*OPRM1*) has been associated with pain sensitivity [10]. The polymorphism (rs1799971) results in a change of amino acid at position 40, in the first exon of the µ-opioid receptor protein from asparagine (Asn) to be replaced by aspartate acid (Asp). One of the SNP consequences points to the increased affinity of altered protein for β-endorphin, which represents a potential mechanism in which this SNP may alter pain sensitivity [11]. The SNP rs4680 is a missense variant and results in a substitution of valine (Val) to methionine (Met) in the product of the gene of catechol-O-methyltransferase (*COMT*) [12]. The alteration causes the production of an enzyme with a lower thermostability (Met/Met genotype), which results in decreased enzyme activity under physiological conditions [13]. Subjects with a Met/Met genotype tend to exhibit higher pain sensitivity when compared to those with the a/Val genotype [14]. Accordingly, the Val158Met rs4680 has been linked to the development of chronic pain [15]. Nevertheless, this association is controversial since the presence of the Met allele has been associated with the risk of developing temporomandibular pain disorders [16], but not migraine [17].

The brain-derived neurotrophic factor (*BDNF*) Val66Met polymorphism (rs6265) induces a substitution at codon 66 in the *BDNF* protein sequence with valine (Val) in the place of methionine (Met), with a consequent reduction of *BDNF* secretion [18]. Previous research has shown how *BDNF* is involved in the crucial process that includes the formation, maturation, and plasticity of neuronal synapses [19] and plays a key role in sensitization and chronic pain [20]. In this direction, studies have shown how the presence of the Met allele is potentially associated with a genetic sensitivity to experimental pain, indicating *BDNF* Val66Met polymorphism is involved in maladaptive neuroplasticity [9]. Minuzzi et al. proposed that the *BDNF* content may serve as a tool predicting worsened prognosis in COVID-19, especially for male patients [21]. These results suggest a potential role of sex in this polymorphism, at least during the acute phase of the disease.

The 5-hydroxytryptamine receptor 1B gene (*HTR1B*) codifies the serotonergic receptor (5-HT1B) involved in various physiological processes such as sleep and pain [22]. Polymorphism rs6296 Val287 results in a synonymous mutation causing a reduction of *HTR1B* mRNA and has been associated with a modulatory effect on exercise-induced hypoalgesia in women with fibromyalgia [8].

For COVID-19 and the mentioned genetic variations, few published studies have focused on their association with post-COVID pain. To date, just a single study has looked into the *COMT* (rs4680) SNP and COVID-19 population suggesting the Met allele of this polymorphism may be associated with a higher impact of COVID-19 across nations [23]; however, no data on long COVID was presented. Thus, it seemed important to evaluate the associations of pain-related SNPs with post-COVID pain to better define the subpopulations more susceptible to experience pain in the long-term after SARS-CoV-2 infection. A better understanding of molecular mechanisms associated with pain at both the acute phase of SARS-CoV-2 infection [24] and also at a post-COVID phase [25] could help clinicians to better manage these symptoms.

The primary aim of this study was to investigate the possible associations between selected SNPs of *OPRM1* (rs1799971), *COMT* (rs4680), *BDNF* (rs6265), and *HTR1B* (rs6296) and the presence of de novo long-term post-COVID pain in previously hospitalized COVID-19 survivors. The secondary aim was to associate the investigated SNPs with sensory-related, cognitive and psychological variables in the individuals with post-COVID pain.

## 2. Methods

### 2.1. Participants

An observational cross-sectional cohort study following *The Strengthening the Reporting of Observational studies in Epidemiology (STROBE)* guidelines [26] was conducted. We recruited individuals who had recovered from acute SARS-CoV-2 infection at three urban hospitals in Spain during the first wave of the pandemic (March–May 2020). Participants should have been hospitalized due to an acute SARS-CoV-2 infection diagnosed by real-time reverse transcription-polymerase chain reaction (RT-PCR) assay of nasopharyngeal or oral swab sample and the presence of consistent clinical and radiological findings at admission. This study was approved by the Institutional Ethics Committees of all institutions involved (URJC0907202015920; HUIL/092-20, HUFA 20/126; HSO25112020). Participants were informed of the study and all provided their written informed consent prior to their inclusion.

### 2.2. Defining Post-COVID Pain

Participants were scheduled for a face-to-face interview for collecting all data. They were asked for the presence of pain symptoms that the patient started to experience after the acute infection. We defined post-COVID pain according to the diagnosis of primary musculoskeletal chronic pain proposed by the International Association for the Study of Pain (IASP) [27]. We focused on those who had developed de novo pain symptoms starting after hospitalization and lasting for at least three consecutive months. Exclusion criteria included: 1, previous history of pain symptoms before the acute infection; and, 2, diagnosis of any pre-existing medical comorbidity better explaining the pain symptoms. In addition, patients were free to report any other symptom that they also experienced after the infection.

### 2.3. DNA Collection and Genotyping

Unstimulated whole saliva samples were collected from each participant into collection tubes according to standardized procedures. Saliva collections were made with patients seated and relaxed, and always during the morning. Participants were asked not to eat, drink or chew gum for 1 h before the sample collection. Those who smoked were asked not to do so from 2 days before collection sampling. The saliva self-collection procedure was non-invasive and immediately after the collection, the samples were centrifuged at 3000 rpm for 15 min to obtain the cell sediment and stored at −20 °C until analysis. We used saliva instead of blood sampling because salivary collection is a suitable non-invasive, stress-free, and ethical assessment method.

Genomic DNA was extracted from 500 mL of saliva using a MagMAX DNA Multi-Sample Ultra 2.0 Kit (Thermo Fisher Scientific Inc., Hemel Hempstead, Hertfordshire, UK) according to the manufacturer’s protocol. We automatically extracted DNA using the King Fisher Flex purification robot (Thermo Fisher, Waltham, MA, USA). The resulting DNA was assessed for purity and concentration using Quant-iT PicoGreen dsDNA reagent” (Thermo Fisher). The resulting DNA was diluted to 5 ng/μL, using 1 × Tris-EDTA (TE) buffer (Sigma-Aldrich, Dorset, Gillingham, UK). The qPCR reaction mixtures of 10 uL contained a total of 10 ng gDNA as a PCR template, 1× TaqMan Gene Expression PCR Master Mix and 0.6× Genotyping TaqMan-probe assay.

For genotyping the single nucleotide polymorphisms with real-time PCR reaction (RT-PCR) TaqMan Predesigned SNP Genotyping Assays was used (Thermo Fisher Scientific Inc., Hemel Hempstead, Hertfordshire, UK). TaqMan SNP Genotyping Assays use TaqMan 5′-nuclease chemistry for amplifying and detecting specific polymorphisms in purified genomic DNA samples. Each assay allows the genotyping of individuals for a single nucleotide polymorphism (SNP). Real-time PCR plates were run in the Quantstudio 12K Flex System (Thermo Fisher) of Genomics Unit (Madrid Science Park Foundation, Madrid, Spain) under standard running conditions (95° for 10 min and 40 two-step cycles consisting of 95 °C for 15 s and 60 °C for 1 min) and analyzed with Genotyping App of Thermo Fisher Cloud. Identification of each of the three possible variants of each SNP was conducted using specific fluorescent dyes.

The possible variants of the *OPRM1* (rs1799971) lead to the following genotypes: Asn/Asn (A/A), Asn/Asp (A/G) or Asp/Asp (G/G). The results were derived from a A→G substitution at the following sequence: GGTCAACTTGTCCCACTTAGATGGC [A/G] ACCTGTCCGACCCATGCGGTCCGAA.

The possible variants of the *COMT* (rs4680) lead to the following three genotypes: Val/Val (G/G), Val/Met (G/A), Met/Met (A/A). The results were derived from A→G substitution at the following sequence: CCAGCGGATGGTGGATTTCGCTGGC [A/G] TGAAGGACAAGGTGTGCATGCCTGA.

The possible variants of the *BDNF* (rs6265) lead to the following genotypes: C/C, C/T or T/T. The results were derived from a C→T substitution at the sequence: TCCTCATCCAACAGCTCTTCTATCA [C/T] GTGTTCGAAAGTGTCAGCCAATGAT.

The possible variants of the *HTR1B* (rs6296) lead to the following genotypes: C/C, C/G or G/G. The results were derived from a C→G substitution at the following sequence: CGGAGACTCGCACTTTGACTTGGTT [C/G] ACATACACAGGAGATCCGGATTCGC.

### 2.4. Post-COVID Pain Featuring

A structured questionnaire including clinical data about pain and several patient-reported outcome measures (PROMs) was completed. The intensity (numerical pain rating scale, NPRS, 0–10) and duration of pain symptoms data were collected. The PROMs evaluated sensory-related (i.e., sensitization-associated symptoms, neuropathic features), cognitive (i.e., catastrophism, kinesiophobia) and psychological (i.e., anxiety and depressive levels, sleep quality) variables.

Sensory-related variables: The presence of sensitization-associated symptoms was evaluated using the Central Sensitization Inventory (CSI, 25 items, 5-point Likert scale, total score 0–100) [28]. The presence of neuropathic pain features was evaluated with the use of the self-report Leeds Assessment of Neuropathic Symptoms (S-LANSS, 7 items, score 0–24) scale [29].

Cognitive variables: Pain catastrophism and kinesiophobia levels were assessed with the Pain Catastrophizing Scale (PCS, 13 items, rated from 0: never to 4: always, total score 0–52) [30] and the 11-item Tampa Scale Kinesiophobia Scale (TSK-11, 11 items, rated from 1: complete disagreement to 4: complete agreement, total score 11–44) [31].

Psychological variables: Anxiety and depressive levels were assessed with the Hospital Anxiety and Depression Scale (HADS-A, HADS-D, 7 items each, score 0–21 points) [32]. Sleep quality was assessed with the Pittsburgh Sleep Quality Index (PSQI, 19 items, rated from 0 to 3, score 0–21 points) [33].

### 2.5. Statistical Analysis

All analyses were performed using SPSS Statistics, version 25.0. Data were reported as mean ± standard deviation (SD). For all inferences, 2-tailed tests were used and a *p*-value of <0.05 was considered significant. Genotype frequencies were analyzed with the Fisher exact test, and chi-squared (χ^2^) tests were used to assess deviations from Hardy–Weinberg equilibrium. Similarly, differences in genotype frequencies between males and females (sex perspective) in the total sample in the subgroup of patients with post-COVID was also assessed with the Fisher exact test and chi-squared (χ^2^) tests. The Shapiro–Wilk test was used to assess the assumption of normality, and when appropriate to use nonparametric tests. Finally, a one-way ANOVA was used to compare sensory-related, cognitive and psychological variables according to the genotypes of each SNP (*OPRM1* rs1799971, *COMT* rs4680, *BDNF* rs6265, *HTR1B* rs6296) in the group of patients with post-COVID pain symptoms. Post hoc analyses comparisons were conducted with the Tukey test.

## 3. Results

Three hundred and fifty (*n* = 350) previously hospitalized COVID-19 patients were randomly invited to participate in the study. Fifty-seven (16%) were excluded: refused to attend the face-to-face interview (*n* = 30), previous diagnosis of fibromyalgia syndrome (*n* = 20), pain from neurological origin (*n* = 5) and pregnancy (*n* = 2). Accordingly, a total of 293 (49.5% female, mean age: 55.6 ± 12.9 years) fulfilled all inclusion criteria, agreed to participate, were included, and assessed 17.8 ± 5.2 months after hospital discharge. One hundred and seventeen (*n* = 39.9%) patients fulfilled the criteria for musculoskeletal post-COVID pain. Table 1 summarizes the demographic data, previous co-morbidities and other post-COVID symptoms in both groups according to the presence or absence of post-COVID pain.

From each saliva sample, DNA was isolated and amplified; however, two samples were compromised during genotyping analysis for the investigated SNPs and therefore excluded. In addition, it was not possible to determine the genotype of some SNPs in another two samples leading to the following numbers on each SNP: *OPRM1* rs1799971 (*n* = 291), *COMT* rs4680 (*n* = 289), *BDNF* rs6265 (*n* = 290), and *HTR1B* rs6296 (*n* = 290).

The genotype distributions did not deviate from those expected based on the Hardy–Weinberg equilibrium. The distribution of the genotypes of any SNP was not significantly different (*OPRM1* rs1799971, *p* = 0.178; *COMT* rs4680, *p* = 0.509; *BDNF* rs6265, *p* = 0.930; *HTR1B* rs6296, *p* = 0.190) between individuals with and without post-COVID pain (Table 2). Overall, the distribution of the genotypes of any SNP was not significantly different (*OPRM1* rs1799971, *p* = 0.258; *COMT* rs4680, *p* = 0.284; *BDNF* rs6265, *p* = 0.326; *HTR1B* rs6296, *p* = 0.494) between males and females. However, analyzing the effect of sex in those individuals with post-COVID pain, a significant effect of sex was observed for the *BDNF* rs6265 (*p* = 0.01) polymorphism, but not for the other SNPs (*OPRM1* rs1799971, *p* = 0.517; *COMT* rs4680, *p* = 0.580; *HTR1B* rs6296, *p* = 0.407): a greater proportion of males (*n* = 4/43, 9%) had the T/T allele when compared with females (*n* = 1/73, 1.3%).

The mixed-model ANOVA did not reveal significant differences in clinical, sensory-related, cognitive and psychological variables depending on the genotype for any SNP *OPRM1* rs1799971 (Table 3), *COMT* rs4680 (Table 4), *BDNF* rs6265 (Table 5), and *HTR1B* rs6296 (Table 6).

## 4. Discussion

In the last decade, researchers have looked thoroughly into the associations of SNP and the stratification of pain perception in healthy populations under physiological conditions or affected by chronic pain conditions such as fibromyalgia or cancer pain and for vulnerability to develop chronic pain. To the best of our knowledge, this is the first study looking into the potential correlations between specific pain features in previously hospitalized COVID-19 survivors with post-COVID pain and selected, specific pain-related SNPs. The current study did not reveal significant differences in the presence of *OPRM1* (rs1799971), *COMT* (rs4680), *BDNF* (rs6265), and *HTR1B* (rs6296) SNPs between previously hospitalized COVID-19 survivors with and without post-COVID pain. Additionally, none of the analyzed pain-related polymorphisms were related to any other features such as sensory-related, cognitive and psychological variables.

### 4.1. OPRM1 (rs1799971) Polymorphism

In the current study, the alteration A118G for the *OPRM1* gene, which results in the modification Asn40Asp, showed no differences between subjects with and without post-COVID pain, and therefore with different pain features. Previous research has highlighted how the A118G SNP in healthy individuals with the presence of the Asp allele reported lower sensitivity to pressure pain compared with the Asn allele [11]. In fact, the presence of the G allele in this SNP has been associated with a poor response to opioids in people with cancer pain [34]. The mechanism behind these alterations is related to alteration of the µ-opioid receptor, reducing or eliminating a putative site for N-linked glycosylation, a relevant process in protein folding, stability, localization, and trafficking; affecting the process of ligand-receptor binding [34]. This process may lead to a potentially altered interaction with β-endorphins, which has been shown in vitro, and alter the response to analgesics in cancer patients [34,35]. In our study, no significant association between SNP rs1799971 and the presence of post-COVID pain was found. It is possible that there is a potential influence of other SNPs associated with different opioid receptors in post-COVID pain and clinical, sensory-related, cognitive and psychological variables in people with long COVID.

### 4.2. COMT (rs4680) Val158Met Polymorphism

No association between the Val158Met SNP of the *COMT* gene and post-COVID pain was found. The COMT is one of the enzymes that inactivates catecholamines, and the SNP rs4680 is an abundant functional polymorphism of the *COMT* that codes the substitution of valine (Val) by methionine (Met) at codon 158. This alteration results, for the Val allele carriers, in an increased degradation of dopamine, norepinephrine, and epinephrine and thus a reduction of these neurotransmitters at synaptic receptors [36]. This unavailability causes the expression of µ-opioid receptors involved in the response to pain stimuli [37]. Thus, single base alterations of *COMT* have been hypothesized to be involved in chronic pain conditions; however, current knowledge does not support this association in widespread musculoskeletal pain conditions [38,39]. Similarly, no association between the Val158Met and the risk of suffering fibromyalgia [40], a condition which is currently being compared with post-COVID pain [41], has been identified either. Nevertheless, preliminary data suggest that the Met allele of this SNP, although not associated with the presence of the condition, has been associated with worse clinical expression of pain perception in women with fibromyalgia [42]. We also did not observe this association in our sample of individuals with post-COVID pain. The only study investigating the role of the rs4680 SNP and COVID-19 observed an association of the Met allele with a higher impact of COVID-19 [24], but no data on long COVID was included.

### 4.3. BDNF (rs6265) Val66Met Polymorphism

The current study also looked into another well-known pain-associated polymorphism, Val66Met (rs6265) of the *BDNF*, which results in reduced secretion of the *BDNF* protein and impaired *BDNF* signaling [43]. BDNF, a neurotrophin involved in the growth, differentiation and survival process of both the peripheral and central neurons, is released when nociceptors are activated, and its action has been associated with the activation of nociceptive pathways. Previous research points at a potential implication in depression and also a potential role in pain mechanisms [44] of the *BDNF* Val66Met polymorphism. However, in this case previous results do not confirm a direct implication of the *BDNF* Val66Met polymorphism in the pain process. For instance, Vossen et al. did not find differences in cortical pain processing between those carriers of the Met allele for *BDNF* [7] whereas Yamada et al. found no association between the *BDNF* Val66Met polymorphism with the clinical and biopsychosocial features in patients with chronic low back pain [43]. In the current study, the absence of an association between Val66Met polymorphism, pain, and clinical, sensory-related, cognitive and psychological variables was also confirmed for a population with long-term post-COVID pain. Nevertheless, we observed a potential effect of male sex in the subgroup of COVID-19 survivors with post-COVID pain, in agreement with correlation proposed by Minuzzi et al. [21] for the acute phase of infection. Due to the small sample of males included in our study, this assumption should be considered with caution at this stage.

### 4.4. HTR1B (rs6296) Val287 Polymorphism

The *HTR1B* (rs6296) polymorphism, involved in the regulation of serotonin levels, was not significantly associated in individuals with post-COVID pain either. This polymorphism has been found to be correlated with suicidal behaviors [45]; however, its role in pain modulation is less clear. For instance, this SNP did not have a direct effect on its own on exercise-induced hypoalgesia in women with fibromyalgia [8]. Even though the potential involvement of this receptor could lead to altered pain perception, no significant association of this SNP with clinical, psychological and pain was featured. In fact, we did not find an association of this SNP with anxiety or depressive levels in our cohort of long COVID patients; however, we do not currently know if this SNP can be associated with other mental disorders, such as post-traumatic stress disorder (PTSD), observed in patients with long COVID.

### 4.5. Limitations

First, only previously hospitalized COVID-19 survivors were included and the role of the investigated SNPs in non-hospitalized cohorts is not currently know. Second, the sample size could be considered small, particularly for detecting sex-linked differences. Studies including larger cohorts are needed to confirm or refute the current exploratory study. Third, we only analyzed four selected SNPs commonly shown to be associated with pain processing. It is possible that biomarkers related to different aspects of the immune inflammatory regulation could reveal other results. Finally, variants in the regulatory regions might change the expression of pain-related genes including those that were analyzed in the current study.

## 5. Conclusions

This exploratory study showed that four polymorphisms associated with pain experience (SNPs of *OPRM1* (*rs1799971*), *COMT* (*rs4680*), *BDNF* (*rs6265*), and *HTR1B* (*rs6296*)), did not appear to predispose for developing post-COVID pain in previously hospitalized COVID-19 survivors. Furthermore, these SNPs were not found to be related to post-COVID sensory-related, psychological or cognitive variables. Cohort studies investigating other specific polymorphisms and deeply analyzing sex-linked differences should be initiated to understand the underlying pathophysiology of post-COVID pain. In fact, only coding variants were included in this study and the potential association of the studied genes with post-COVID pain through their non-coding regulatory variants cannot be ruled out.

## Figures and Tables

**Table 1:** Demographic, previous co-morbidities and other post-COVID symptoms in COVID-19 survivors according to the presence or absence of post-COVID pain.

	Post-COVID Pain (*n* = 117)	No Post-COVID Pain (*n* = 176)
Age, mean (SD), years	56.0 (12.5)	57.5 (13.5)
Gender, female *n* (%) *	74 (63.2%)	81 (46.1%)
Weight, mean (SD), kg.	80.8 (17.9)	80.8 (15.8)
Height, mean (SD), cm.	168 (8.5)	168 (9.5)
Medical co-morbidities, *n* (%)		
Hypertension	36 (30.7%)	54 (30.6%)
Diabetes	13 (11.1%)	16 (9.1%)
Cardiovascular diseases	6 (5.1%)	12 (6.8%)
Asthma	12 (10.2%)	17 (9.6%)
Obesity	30 (25.6%)	40 (22.7%)
Chronic obstructive pulmonary disease	2 (1.7%)	5 (2.8%)
Rheumatological diseases	4 (3.4%)	6 (3.4%)
Other (cancer, kidney disease)	21 (17.9%)	31 (17.6%)
Post-COVID symptoms, *n* (%)		
Fatigue *	92 (78.6%)	92 (52.3%)
Dyspnea	19 (16.2%)	22 (12.5%)
Skin rashes	35 (29.9%)	43 (24.4%)
Memory loss	24 (20.5%)	34 (19.3%)
Concentration loss	16 (13.7%)	18 (10.2%)
Cognitive blunting–brain fog	15 (18.8%)	20 (11.3%)
Gastrointestinal disorders	8 (6.8%)	15 (8.5%)
Ocular disorders	7 (6.0%)	10 (5.7%)
Ageusia/hypogeusia	5 (4.2%)	7 (3.9%)
Anosmia/hyposmia	6 (5.1%)	8 (4.5%)

*n*: number; SD: standard deviation; * statistically significant differences (*p* < 0.001).

**Table 2:** Distribution of genotypes of the pain polymorphisms assessed in people with long COVID.

	Post-COVID Pain	No Post-COVID Pain	*p* Value
*OPRM1 rs1799971* (*n* = 291)			
Asn/Asn (*n* = 202)	78 (66.6%)	124 (71.2%)	χ^2^ = 3.453; *p* = 0.178
Asn/Asp (*n* = 82)	38 (32.5%)	44 (25.3%)	
Asp/Asp (*n* = 7)	1 (0.09%)	6 (3.5%)	
*COMT rs4680* (*n* = 289)			
Val/Val (*n* = 48)	16 (13.6%)	32 (18.6%)	χ^2^ = 1.352; *p* = 0.509
Val/Met (*n* = 144)	59 (50.4%)	85 (49.4%)	
Met/Met (*n* = 97)	42 (36.0%)	55 (32.0%)	
*BDNF rs6265* (*n* = 290)			
C/C (*n* = 197)	80 (68.9%)	117 (67.2%)	χ^2^ = 0.145; *p* = 0.930
C/T (*n* = 81)	31 (26.7%)	50 (28.7%)	
T/T (*n* = 12)	5 (4.4%)	7 (4.1%)	
*HTR1B rs6296* (*n* = 290)			
C/C (*n* = 157)	57 (48.7%)	100 (57.8%)	χ^2^ = 3.321; *p* = 0.190
C/G (*n* = 113)	53 (45.3%)	60 (34.7%)	
G/G (*n* = 20)	7 (6.0%)	13 (7.5%)	

**Table 3:** Differences in clinical, sensory-related, cognitive and psychological variables in subjects with post-COVID pain depending on the µ-opioid receptor gene (*OPRM1*) polymorphism (*rs1799971*).

	Asn/Asn (*n* = 78)	Asn/Asp (*n* = 38)	Asp/Asp (*n* = 1)
Demographic Features			
Age (years)	54.7 ± 12.0	55.5 ± 12.8	62
Height (m)	1.65 ± 0.1	1.64 ± 0.2	1.65
Weight (kg)	80.5 ± 17.2	81.8 ± 19.5	80.0
Clinical Features			
Pain intensity (NPRS, 0–10)	5.5 ± 1.7	5.9 ± 1.75	5.2
Post-COVID Symptoms (months)	17.6 ± 5.3	17.9 ± 4.5	18.0
Sensory-Related Variables			
Central Sensitization Inventory (0–100)	35.5 ± 18.2	34.5 ± 18.7	42.0
S-LANSS (0–24)	5.6 ± 5.7	10.2 ± 13.3	4.0
Cognitive Variables			
Pain Catastrophizing Scale (0–52)	8.9 ± 10.1	7.0 ± 7.9	7.0
Tampa Scale for Kinesiophobia (0–44)	23.4 ± 8.8	22.4 ± 8.6	30.0
Psychological Variables			
HADS-A (0–21)	3.7 ± 3.9	3.5 ± 4.5	3.3
HADS-D (0–21)	3.0 ± 3.8	2.9 ± 3.9	3.0
PSQI (0–21)	6.5 ± 3.7	6.55 ± 4.1	6.4

NPRS: numerical pain rate scale; HADS: Hospital Anxiety and Depression Scale (A: anxiety; D: depression); S-LANSS: self-reported version of the Leeds Assessment of Neuropathic Symptoms and Signs; PSQI: Pittsburgh Sleep Quality Index.

**Table 4:** Differences in clinical, sensory-related, cognitive and psychological variables in subjects with post-COVID pain depending on the catechol-O-methyltransferase gene (*COMT*) Val158Met polymorphism (*rs4680*).

	Val/Val (*n* = 16)	Val/Met (*n* = 59)	Met/Met (*n* = 42)
Demographic Features			
Age (years)	54.5 ± 11.5	56.0 ± 12.1	54.1 ± 13.2
Height (m)	1.64 ± 0.09	1.69 ± 0.1	1.65 ± 0.08
Weight (kg)	81.8 ± 12.9	82.9 ± 14.2	80.9 ± 13.9
Clinical Features			
Pain intensity (NPRS, 0–10)	5.1 ± 1.8	5.9 ± 1.7	5.5 ± 1.6
Post-COVID symptoms (months)	17.5 ± 5.4	17.4 ± 5.2	18.1 ± 5.0
Sensory-Related Variables			
Central sensitization inventory (0–100)	32.3 ± 19.9	35.9 ± 16.7	40.9 ± 19.3
S-LANSS (0–24)	5.9 ± 7.5	6.6 ± 6.2	8.1 ± 12.7
Cognitive Variables			
Pain catastrophizing scale (0–52)	7.9 ± 9.0	8.6 ± 8.7	8.0 ± 10.8
Tampa Scale for Kinesiophobia (0–44)	23.7 ± 7.2	23.9 ± 8.2	22.9 ± 10.0
Psychological Variables			
HADS-A (0–21)	3.5 ± 4.4	4.0 ± 4.1	4.2 ± 4.0
HADS-D (0–21)	3.25 ± 4.9	3.2 ± 3.9	3.3 ± 3.1
PSQI (0–21)	6.1 ± 4.4	6.9 ± 4.0	6.0 ± 3.8

NPRS: numerical pain rate scale; HADS: Hospital Anxiety and Depression Scale (A: anxiety; D: depression); S-LANSS: self-reported version of the Leeds Assessment of Neuropathic Symptoms and Signs; PSQI: Pittsburgh Sleep Quality Index.

**Table 5:** Differences in clinical, sensory-related, cognitive and psychological variables in individuals with post-COVID pain depending on the brain-derived neurotrophic factor (*BDNF*) Val66Met polymorphism (*rs6265*).

	C/C (*n* = 80)	C/T (*n* = 31)	T/T (*n* = 5)
Demographic Features			
Age (years)	54.5 ± 12.7	57.0 ± 12.2	55.0 ± 9.3
Height (m)	1.66 ± 0.08	1.67 ± 0.09	1.70 ± 0.12
Weight (kg)	80.0 ± 18.8	80.1 ± 16.2	84.2 ± 13.8
Clinical Features			
Pain intensity (NPRS, 0–10)	5.6 ± 1.7	5.7 ± 1.6	5.5 ± 0.7
Post-COVID symptoms (months)	17.5 ± 5.3	17.7 ± 5.1	19.0 ± 2.8
Sensory-Related Variables			
Central sensitization inventory (0–100)	37.5 ± 18.6	31.4 ± 17.9	37.6. ± 14.0
S-LANSS (0–24)	6.4 ± 5.8	9.2 ± 6.8	7.2 ± 5.7
Cognitive Variables			
Pain catastrophizing scale (0–52)	9.4 ± 10.3	6.5 ± 6.9	9.4 ± 2.6
Tampa Scale for Kinesiophobia (0–44)	23.9 ± 9.4	21.5 ± 7.0	23.0 ± 7.8
Psychological Variables			
HADS-A (0–21)	3.9 ± 4.3	3.1 ± 3.5	3.6 ± 3.2
HADS-D (0–21)	2.8 ± 3.9	3.3 ± 4.0	3.25 ± 2.0
PSQI (0–21)	6.3 ± 3.9	6.9 ± 3.7	6.1 ± 3.2

NPRS: numerical pain rate scale; HADS: Hospital Anxiety and Depression Scale (A: anxiety; D: depression); S-LANSS: self-reported version of the Leeds Assessment of Neuropathic Symptoms and Signs; PSQI: Pittsburgh Sleep Quality Index.

**Table 6:** Differences in clinical, sensory-related, cognitive and psychological variables in individuals with post-COVID pain depending on the 5-hydroxytryptamine receptor 1B gene (*HTR1B*) Val287 polymorphism (*rs6296*).

	C/C (*n* = 57)	C/G (*n* = 53)	G/G (*n* = 7)
Demographic Features			
Age (years)	56.1 ± 12.7	55.0 ± 12.3	52.9 ± 12.5
Height (m)	1.66 ± 0.09	1.68 ± 0.16	1.67 ± 0.10
Weight (kg)	81.1 ± 15.7	80.8 ± 16.0	78.5 ± 18.1
Clinical Features			
Pain intensity (NPRS, 0–10)	5.4 ± 1.7	5.9 ± 1.5	5.2 ± 2.5
Post-COVID symptoms (months)	18.0 ± 5.0	17.1 ± 5.5	19.0 ± 3.9
Sensory-Related Variables			
Central sensitization inventory (0–100)	35.25 ± 19.3	35.0 ± 17.5	39.00 ± 18.5
S-LANSS (0–24)	7.9 ± 11.4	6.0 ± 6.1	8.4 ± 7.7
Cognitive Variables			
Pain catastrophizing scale (0–52)	7.8 ± 7.6	8.4 ± 10.8	10 ± 11.9
Tampa Scale for Kinesiophobia (0–44)	23.9 ± 8.3	21.9 ± 9.4	26.6 ± 4.8
Psychological Variables			
HADS-A (0–21)	3.5 ± 3.9	3.8 ± 4.2	4.0 ± 4.8
HADS-D (0–21)	2.7 ± 3.5	3.1 ± 4.1	3.4 ± 4.2
PSQI (0–21)	6.5 ± 3.7	6.3 ± 4.0	6.6 ± 3.8

NPRS: numerical pain rate scale; HADS: Hospital Anxiety and Depression Scale (A: anxiety; D: depression); S-LANSS: self-reported version of the Leeds Assessment of Neuropathic Symptoms and Signs; PSQI: Pittsburgh Sleep Quality Index.

## Data Availability

All data derived from this study are presented in the text.
